# Sub-national perspectives on the implementation of a national community health worker programme in Gauteng Province, South Africa

**DOI:** 10.1136/bmjgh-2019-001564

**Published:** 2019-11-28

**Authors:** Shehnaz Munshi, Nicola J Christofides, John Eyles

**Affiliations:** 1 Centre for Health Policy, School of Public Health, Faculty of Health Sciences, University of the Witwatersrand, Johannesburg, Gauteng, South Africa; 2 School of Public Health, Faculty of Health Sciences, University of the Witwatersrand, Johannesburg, Gauteng, South Africa; 3 School of Geography and Earth Sciences, McMaster University, Hamilton, Ontario, Canada

**Keywords:** qualitative study, public health, health systems, health policy, health education and promotion

## Abstract

**Introduction:**

In 2011, in line with principles for Universal Health Coverage, South Africa formalised community health workers (CHWs) into the national health system in order to strengthen primary healthcare. The national policy proposed that teams of CHWs, called Ward-based Primary Healthcare Outreach Teams (WBPHCOTs), supervised by a professional nurse were implemented. This paper explores WBPHCOTs’ and managers’ perspectives on the implementation of the CHW programme in one district in South Africa at the early stages of implementation guided by the Implementation Stages Framework.

**Methods:**

We conducted a qualitative study consisting of five focus group discussions and 14 in-depth interviews with CHWs, team leaders and managers. A content analysis of data was conducted.

**Results:**

There were significant weaknesses in early implementation resulting from a vague national policy and a rushed implementation plan. During the installation stage, adaptations were made to address gaps including the appointment of subdistrict managers and enrolled nurses as team leaders. Staff preparation of CHWs and team leaders to perform their roles was inadequate. To compensate, team members supported each another and assisted with technical skills where they could. Structural issues, such as CHWs receiving a stipend rather than being employed, were an ongoing implementation challenge. Another challenge was that facility managers were employed by the local government authority while the CHW programme was perceived to be a provincial programme.

**Conclusion:**

The implementation of complex programmes requires a shared vision held by all stakeholders. Adaptations occur at different implementation stages, which require a feedback mechanism to inform the implementation in other settings. The CHW programme represented a policy advance but lacked detail with respect to human resources, budget, supervision, training and sustainability, which made it a difficult furrow to plough. This study points to how progressive reform remains fraught without due attention to the minutiae of practice.

**Key messages**

**What is already known?**

- There are often implementation challenges on the ground when new top-down policies are implemented; the community health worker (CHW) programme implemented in South Africa as part of health sector reform was no different. New programmes need to be integrated in the health system and embedded in the communities they serve.

**What are the new findings?**

- Gaps in the fit between the programme and the organisation of the health system create implementation challenges.
- Applying the Implementation Stages Framework highlighted how gaps in the exploration stage affected implementation of the CHW programme during the installation phase.
- The Implementation Stages Framework recognises that there is movement back and forth between the stages but did not account for the adaptations that occurred on the ground to compensate for gaps and challenges in implementation.
- A thoughtful and capable manager at the district level facilitated the implementation of the new programme, but was insufficient to overcome structural and microlevel challenges.

**What do the new findings imply?**

- A mechanism needs to be established so that adaptations on the ground can be shared with other districts and settings where a programme is being implemented.
- Our study shows that detailed planning for implementation of new policies is critical if programmes are to result in successful sustainable systemic change in community health systems.

## Introduction

Community health workers (CHWs) remain a significant cadre in National Health Systems^1–5^ and are integral to achieving the sustainable development goal of universal health coverage (UHC) in low- and middle-income countries.^3 4 6–8^ Recognition of the contribution of CHWs to improving health outcomes has shifted global thinking towards formalising them into the health workforce. In this way, CHWs are recognised as a potential solution for the human resource for health crisis and have become essential parts of globally designed and country-specific community-based programmes to address HIV/AIDs, tuberculosis (TB) and maternal and child health, among other health issues.^9–11^ Yet, the expansion of national CHW programmes following the Alma Ata Declaration of 1978 had variable success and encountered substantive problems such as poor management, unrealistic expectations, poor planning, resourcing and overwhelming workloads.^9 12^

In low- and middle-income countries, there has been a revitalisation of primary healthcare (PHC) and with it renewed attention on CHW programmes and community health systems.^13–16^ To ensure success, there is a need to shift thinking as CHWs are part of the health system, embedded in the community, and require a comprehensive approach to financing, human resources and governance to ensure integration with the formal health system.^7 15 17 18^ Critical to realising the potential of CHW programmes is meaningful involvement of actors such as local political structures, civic groups and faith-based organisations.^4^

In 2011, in line with South Africa’s plans for universal healthcare, a policy of ‘re-engineering PHC’ formalised CHWs into the National Health System.^19^ The policy reform aimed to strengthen PHC through new programmes including integrating CHWs to work in Ward-based Primary Healthcare Outreach Teams (WBPHCOT).^19 20^ This paper focuses on the implementation of the CHW programme or WBPHCOTs.

The national policy proposed that WBPHCOTs should be comprised of six generalist CHWs, led by a PHC facility-based professional nurse, called a team leader. WBPHCOTs were expected to work closely with health promotors and environmental health officers. Each team was assigned to 250 households in the catchment area of a local PHC facility. Facility managers of either clinics or community health centres provided supervision of WBPHCOT team leaders.^19^

The intention in the policy was that CHWs carry out promotive and preventive care, including maternal and child health, non-communicable diseases, HIV and TB, an expansion of their previous roles in non-governmental organisations as mainly TB or HIV care workers.^21^ The policy required CHWs to be formalised into a integrated, comprehensive PHC outreach programme.^22^ Furthermore, they had to operate as part of an integrated service with sectors such as social development, home affairs and education to address barriers to healthcare access.^19^

Alongside the policy, the National Department of Health provided a training curriculum for CHWs and monitoring and evaluation indicators. However, implementation was left to provincial discretion and there were no detailed guidelines for how this should happen.^19 23^ There were variations in the implementation between provinces.^24–33^ In some provinces, universities and non-governmental organisations were contracted as implementing partners, while in others the province/districts managed the process. By mid-2014, at least 1063 WBPHCOTs were active nationwide.^34^ At the time of this study, Gauteng Province (where our study was located) had established 50 WBPHCOTs.^35^

### Analytic approach: implementation framework

**Figure 1:**
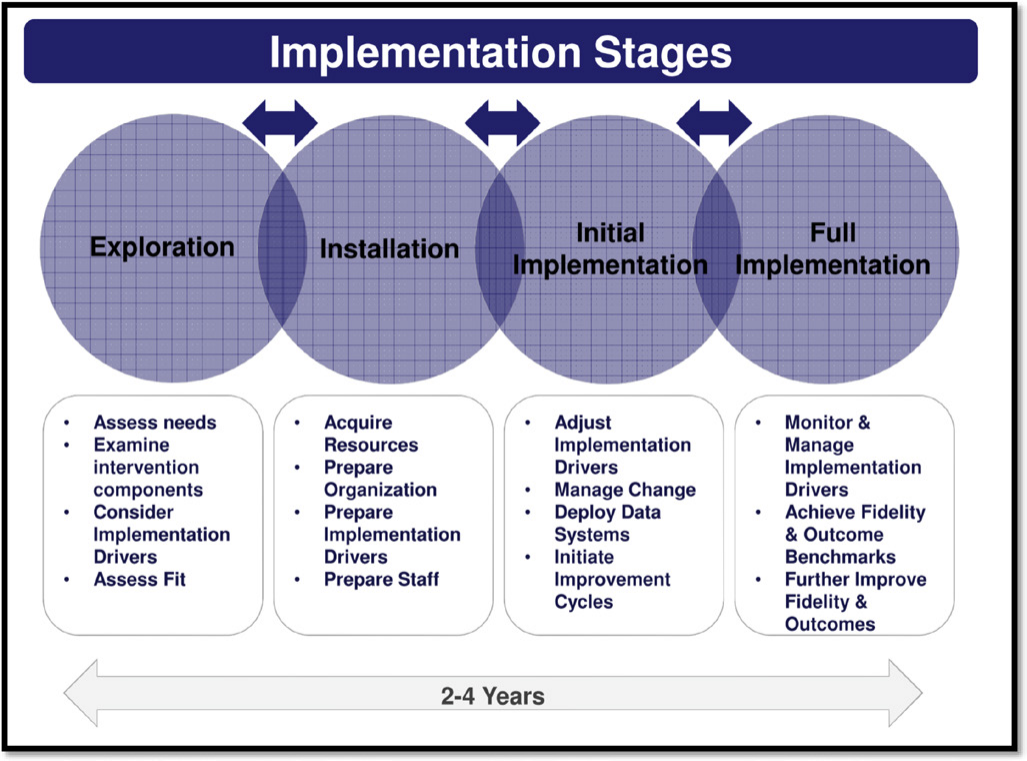
Implementation Stages Framework from Duda (2013).^40^

Implementation frameworks can assist in assessing the multiple factors that influence successful policy implementation. This study employed the National Implementation Research Network’s Implementation Stages Framework (ISF) to analyse the implementation of the WBPHCOT programme. It allowed for the detailed examination of the specific processes and tasks required in stages of implementation.^36–39^ There are four stages in the ISF: exploration, installation, initial implementation and full implementation (figure 1).^40^ The framework acknowledges some back and forth movement between the stages.

The first ‘exploration stage’ assesses the feasibility and readiness of the institution for implementation.^41^ Organisational readiness is described as a shared psychological state characterised by collective commitment to change and is often a precursor to successful organisational change.^42–44^ Steps include creating implementation teams, conducting a context-based need assessment; and ensuring the intervention is ‘usable’, that is developed based on a set of values and evidence, articulates critical functions of the intervention and outlines of how each function is operationalised.^38^

The second ‘installation stage’ involves preparation of the organisation and staff. Resources are acquired or developed to fully and effectively engage in the new ways of working. Preparation of the essential infrastructural elements needed for effective implementation, such as the competence, organisational and leadership drivers is put in place.^38^

During the third, ‘initial implementation stage’, staff members attempt to use their newly learnt skills and ways of working. Quality improvement cycles are initiated to make sure the new policy is making a difference and is relevant.^38^ The third and final ‘full implementation’ stages are outside the scope of this study, which was conducted approximately 2 years after the introduction of the new policy.

The paper analyses the implementation of South Africa’s CHW programme in a large urban/peri-urban district using the ISF. It explores implementation processes and activities during the early stages of implementation (the exploration and installation stages), 8 to 24 months after the teams were established.

## Methods

### Study setting and participants

An exploratory qualitative study was conducted between September 2014 and March 2015. One of the five districts located in the Gauteng Province of South Africa was purposively selected for this study. Gauteng is the wealthiest of the nine provinces in South Africa and is the economic hub. It is characterised by extreme income inequality with both wealthy and poverty-stricken areas.^45^

The selected district has three subdistricts and is made up of urban, peri-urban and semirural areas with a population of approximately 3.18 million people and an annual population growth rate of 2.47% (2001–2011). It is characterised by ethnic diversity due to in-migration from more rural provinces in South Africa and some cross-border migration mainly from neighbouring countries.^45^

Two health authorities govern healthcare provision in the district. The local government authority, funded by the municipality, is the main provider of PHC and is responsible for health promotion and disease prevention services.^46^ These services are delivered through 66 PHC clinics. The provincial health authority is responsible for seven community health centres, which are larger 24-hour maternal and obstetric units that receive referrals from municipal clinics.

Six WBPHCOTs were selected with the assistance of a public health specialist in charge of implementation. The teams were selected if they had been operating for 6 months or more, and the CHWs and team leaders consented to participate. They were linked to a health facility, either a PHC clinic or a community health centre. Five focus group discussions (FGDs), involving 51 CHWs, and 14 in-depth interviews were conducted with facility-based staff, managers and team leaders, all of whom were directly involved with the WBPHCOTs. One FGD did not take place due to lack of availability of the team. Key topics that guided interviews included perceptions of roles, experiences of implementation, challenges to, and enablers of, implementation, relationships within the health facility, team dynamics and areas for improvements.

All interviews and FGDs were audio-recorded with consent. Detailed field notes were taken during site visits, based on observations of facilities, catchment areas and shadowing of CHWs’ interactions when conducting home visits.

Trustworthiness was assured through intercoder agreement obtained by the first author (SM) developing the initial coding schema and co-authors applying it to a subset of transcripts. Preliminary findings were presented back to the district. The field notes provided a rich description of the context and allowed for triangulation of findings from in-depth interviews and FGDs.

### Data analysis

All transcripts and field notes were imported into MAXQDA 12, a qualitative data analysis software programme. Content analysis involved coding text segments across all transcripts and field notes.^47^ Some topic or deductive codes such as implementation challenges were based on the questions asked in the interview while other codes were developed inductively from a deep reading of the transcripts. The coding schema with a definition of each code was developed.^47^ Codes were later categorised and themes were developed at a more interpretive level. Field notes helped to further contextualise the themes that were identified in the analysis.^48^

## Results

Given the timing of the study, the programme was immersed in the installation stage of implementation (figure 1).^38 39^ The findings mainly focus on the installation stage and pick up on some exploration stage factors that were not fully in place, reflecting the overlap between these early stages.

The CHW programme was initiated under the leadership of a public health specialist who was committed to implementation. Although she did not have direct access to and control over budgets, she had decision space relating to the organisation of existing resources. At the time of the study, about 2 years after the introduction of the policy, the district had established nine teams, prioritising poorer areas.

The six selected teams had been working for a period between 8 and 24 months. The number of CHWs per team ranged from 7 to 12. Some larger teams accommodated larger catchment areas. Of the 51 CHWs in the study, most (n=41) were women. The majority of CHW were between 20 and 40 years of age (n=43) and the remainder were over 40 years of age.

Of the six team leaders in the study, two were professional nurses and four were enrolled nurses. All team leaders were women. Three facility managers, one non-governmental organisation manager and two subdistrict managers, all women, participated in the study, as well as a male health promotor and family physician.

The six teams were linked to one community health centre and five clinics of varying size. For example, a smaller clinic, located in a peri-urban catchment area, had only two consulting rooms and a waiting room. The FGD was held outside in the yard under a tree behind the clinic where CHWs gather for meetings. Another clinic was located in a large commercial area and had several additional extensions to the buildings to cater to a large and often mobile community.

### Exploration stage

#### Preparation for the CHW programme

Participants reported that the process of implementation ‘was [a] rush[ed] thing’, which occurred almost immediately after the policy was introduced. Some participants reported ‘preparatory work was not appropriately done’ affecting the district’s ability to plan effectively for adequate staffing, resources and infrastructure.

Efforts were made to introduce the CHW programme to the community, but most participants felt these efforts did not reach all relevant stakeholders, including ‘civil society structures and political leadership’. CHWs experienced unrealistic expectations from the communities such as the provision of ‘food parcels’ and ‘fixing their electricity or water’.

The implementation, maybe the preparation [in the district], was not so well done. Some of the challenges in the process of implementation that I found out … in terms of the community involvement, the initial step, community meeting organised where the CHWs [were] supposed to be introduced to the community but the meetings were poorly attended. (Manager 4)

Preparation for the introduction of the CHW programme was perceived to be inadequate at the community level and within the health facilities. CHWs described feeling unwelcome, and reported that they were not always viewed as being part of the staff and felt unrecognised as healthcare providers.

I don’t think we have a relationship here with the clinic. We arrive in the morning, sign the register, if they greet us, they greet us, if they don’t, they don’t. And then we do our thing and leave…We’re not even appreciated, we are not recognised. (FGD1)

CHWs were not allocated a space of their own, instead they were expected to share rooms with nurses, occupy a vacant room or use shared spaces such as boardrooms or waiting areas. Paperwork, such as household registration forms, was stored in passageways, boxes and outbuildings of the health facility.

So now I must go around ask the provincial nurses in the clinic if I can order [daily supplies] for CHWs. But I know that the problem is storage! They can order, but where are they going to keep those things? (Manager 7)

#### Fit of the programme within the context

There was a mismatch between employment expectations of CHWs and the design of the CHW programme. CHWs were employed on a contractual basis, earning a monthly stipend of R 2200 ($159.33). This amount was a flat rate and was not adjusted based on years of experience or prior earnings. CHWs wanted to be incorporated into the health system, with the same benefits afforded to permanent employees such as sick leave and unemployment insurance. They also wanted a clear career path.

There’s nothing. If you are dead, you’re just dead. That’s your own business. No leave. No sick note, no sick leave. If I’m sick they replace me, like if I get TB from a community member, or if I get hurt in a patient’s house or if a dog bites me; even if I get raped, it’s my business. If you’re going to someone’s house it’s a bit risky because you’re going to find many people smoking nyaope (heroine with a mixture substances such as of cannabis, detergents, rat poison and/or ARV’s)^49 50^), so it’s a risk. (FDG3)

### Learning from implementation

Adaptations to the programme occurred during implementation as gaps became apparent. Midway through the study, the public health specialist appointed professional nurses as subdistrict managers, specifically responsible for the CHW programme. These appointments increased support to the teams and liaison with stakeholders to get more buy-in within the health facilities and communities.

My role is to make sure that we implement WBPHCOT teams in all the wards, we have to support the teams that already exist and supervise them and make sure everything goes well. I have to liaise with all the stakeholders that are involved … so that they buy in. (Manager 7)

Team leaders described the subdistrict managers and public health specialist as responsive and ‘always available’ to their needs.

If I am having a problem, I call them [sub-district manager] then I know that the problem will be solved. Unless if they don’t have like powers like the equipment… So it’s easier then to talk to her. She is going to deal with it (Team leader 6).

In addition, the relationships between CHWs and team leaders developed over time and were viewed by participants as positive and based on mutual respect.

I am working with CHWs, they just earn a stipend, but most of them, let me say all of them, they love their job… they want to help, that’s their goal. So when I work with people like those ones, like every time they are having power you know that, everything is going to be ok. Ja I can say so, you know what when you are managing like adults, most of them they are older than me, you need like to respect them. So that they can give you back that respect. So it is all about respecting each other and do the work (Team leader 6).

### Installation stage

At the installation stage, our findings mostly related to staff preparation and how the health system was organised in the district.

#### Selection and training of CHWs

Consistent with the policy, the district selected and recruited home-based carers, TB Direct Observed Treatment Supporter and HIV counsellors from non-governmental organisations to be CHWs from the PHC facility catchment area. CHWs’ knowledge and skills were mixed. Some teams reported assisting each other to ‘fill out the household registration forms’ as the task was challenging for some members.

The district contracted a non-governmental organisation to train CHWs and team leaders. Participants reported attending the 10-day training, but complained that it was too short to cover the manual content and to prepare them adequately for the health issues they saw during household visits. Many CHWs reported returning from training without a clear understanding of standard operating procedures.

We don’t have enough training. When you out in the field, I didn’t know about schizophrenia, umbilical hernia and stuff, but now I’ve heard of those words, but I don’t know what they mean. So really, we should get more training. (FGD1)

At the time of the study, only one team had received the 5 days of practical training. This posed a significant challenge because CHWs in South Africa had no background in maternal and child health or chronic health issues prior to their employment on the WBPHCOT programme.

[Practical training] is so important because CHWs learn important skills. Inside the tool book is where CHWs learn about the antenatal care. They emphasise the visits that CHW must attend, what to do and what to teach women when they are there. (Manager 8)

#### Selection and training of team leaders

The district started implementation of the WBPHCOT programme without having ‘sufficient professional nurses to act as team leaders’. The district adapted by appointing enrolled nurses as team leaders, despite concerns by managers and the family physician about their lack of training and competency to lead WBPHCOTs effectively.

This programme needs somebody who knows midwifery, who can manage pregnant woman, children, who knows how to immunize babies. Immunisation and [antenatal care] are not the role of enrolled nurses. It’s a big challenge because they can’t even mentor CHWs. They can write the stats, but can’t interpret the report at the end of the month. (Manager 8)

One enrolled nurse acknowledged her lack of experience and limited training to be a team leader.

[Nurses in the health facility] were complaining that how can they go and employ an enrolled nurse to lead WBPHCOTS. Because it needs someone who is PHC trained. So normally, if I have challenges that I cant solve, I go to someone who is PHC trained and I ask them what is this and they will explain how [I can] deal with this. (Team leader 1)

Most team leaders also felt they did not have enough training for the CHW programme. Some team leaders sought guidance from senior nurses or subdistrict manager for both administrative and clinical issues.

One-week training for this project is not enough, we need more. I am lucky because if I have a question I call [sub-district manager], “what do I have to fill out on this form”. What about other team leaders? Without support, they can write the wrong information. (Team leaders 1)

#### Undertaking the programme within two jurisdictions

The complex history of having two jurisdictions responsible for PHC delivery in the district created two lines of authority and resulted in some confusion among managers. Some participants expected the province to provide human and material resources because there was an impression that the CHW programme was provincially run. Municipal clinics had a separate budget through the local government authority structures and allocated resources based on their own set of priorities. According to the policy, districts and municipalities were expected to increase efficiencies by redirecting existing human, financial and commodity resources towards PHC re-engineering. However, managers were unsure about the financing of the CHW programme.

Ja, we don’t have budget, I thought maybe they [government] are still planning for [WBPHCOTs], I don’t know. (Manager 8)

The lack of clarity between the two jurisdictions about how the CHW programme fitted in resulted in team leaders having limited access to resources, such as telephones and stationery, procured by the municipality. This negatively affected communication between team leaders and CHWs, especially when CHWs needed guidance during a home visit.

Most of our team leaders are provincial staff so they don’t have the code you need to use the [municipal clinic] phones. (Manager 7)

At a management level, team leaders were expected to carry out tasks outlined in the provincially led CHW programme, while at the same time complying with clinical duties determined by facility managers.

I do have team leaders who are not allowed, maybe they are not given time to function properly because of [staff] shortages, so because it is municipality [clinic], and I am provincially [employed], I cannot force. Sometimes team leaders phone saying she is not coming to the meeting there is a [staff] shortage (Manager 8).

The policy states that facility managers are responsible for integrating CHWs into clinics and providing resources to support outreach activities, yet participants reported varying experiences depending on the facility manager’s understanding and interest in the programme. Some facility managers supported the CHW programme, while others prioritised local government authority’s responsibilities, preventing team leaders from fulfilling their roles effectively.

[A team leader] working at the local government clinic, they don’t allow her to function independently. However, the other team leader is working at a [different local government authority] where the manager is interested in WBPHCOT, so she is doing very well. (Manager 8)

All team leaders reported having clinical responsibilities that prevented them from fulfilling all their WBPHCOT responsibilities.

I [oversee] antenatal care, EPI [Expanded Programme on Immunisation] programme. I attend meetings for maternal issues. Every day I see patients, there is no day that I don’t see patients. (Team leader 3)

#### Mobilising resources

WBPHCOT members felt frustrated by the lack of available resources. CHWs complained that they did not have ‘basic’ equipment such ‘adult kimbi [nappy], linen savers and gloves’. CHWs felt their productivity was hampered because they did not have stationery, identifiable uniforms or cell phones to contact team leaders. Transport emerged as a significant implementation barrier in areas where CHWs had to cover large distances on foot to reach the households in the catchment area. The lack of transportation limited team leaders’ access to the team to conduct supervisory visits.

CHWs didn’t have identification and uniform, so the person threatened to go to the police and get them arrested because of that. So I can say the community is still not clear about what’s happening in the clinic about the PHC outreach team. They don’t understand, even though when we started, we [team leader] go with the people who are in charge, and we got permission from the councillor (Team leader 1).

### Factors supporting implementation: CHW motivation

Despite all the implementation challenges, CHW still felt motivated and committed to the job. CHWs discussed their role with pride and valued the opportunity to make a difference in their communities.

We like our job, only if some of our challenges could be sorted, then it will be even much more easier because it’s a purpose to do that. It’s our passion. We like our job, to communicate, socialize and make a relationship. You know the smile afterwards, It says a lot. We have a relationship with these people. When they say thank you, It says a lot that I’m doing the best job. Or when you find someone sick, you go around with that person until they are better, you see them walking in the street, that’s also a good feeling. We also bond with them. (FGD2)

Despite their own financial strain, CHWs helped community members facing precarious situations. One manager described the resilience and determination of CHWs:

The little money they get, if they get it, it needs to be used for a lot of things. Sometimes they go to a patient, where she’s got no food and they had to make a plan for that. You see? They use their own money for transportation [to work]. They use their own money to help somebody who is there, you see all that. (Manager 2)

## Discussion

This paper details the early implementation of a national WBPHCOT initiative, as part of reforms to strengthen PHC, through investigating CHWs’, team leaders’ and managers’ perspectives on the implementation of the programme in one district in Gauteng Province, South Africa. It adds to the understanding of how implementation of top-down policies happens on the ground, using the ISF, with a focus on the exploration and installation stages.

By conducting the research at a critical time early in the implementation process, unique insights and key lessons from the ISF stages, and how they are related, emerged from our study. We found that most of the implementation gaps that emerged during the installation stage resulted from weaknesses that occurred in the exploration stage. It demonstrated the mismatch between national (central level) intentions and district (local level) implementation.^51^

We found that the CHW programme did not meet fully the criteria described by ISF for a ‘usable innovation’.^38^ Essential functions that frame the innovation were missing, such as how each function was to be operationalised. Implementation was left to the capacity and inclination of provinces or districts to plan and operationalise.^32^ There was inadequate district preparation to ensure readiness at the institutional and community levels. Provinces and districts had to scale up the programme as an unfunded mandate, within existing and already stretched resource envelopes.^23 24 52 53^

A facilitating factor in the implementation of the CHW programme, in the district under study, was the deployment of a public health specialist, who was part of the district public health unit.^54^ She provided the vision, which was aligned to the policy, making her the right person with relevant values to take it on. She began the process of establishing teams, initiating community and health system entry and later appointing subdistrict managers. However, the leadership that she provided did not make up for the lack of fit between the programme and the structural factors in the district.

Weaknesses were identified in micro-level leadership at clinics and community health centres, where levels of support and buy-in from facility managers influenced the experiences of CHWs and the degree of implementation. Even those who supported the programme were not champions, resulting in low levels of willingness to prioritise and reallocate resources (staff and space) to CHWs.^32 51^ Other studies have shown that forging a collective vision and having a champion at the very local level can be an enabler in the implementation process.^32 33 55 56^

Similar to North West Province and a district in the Western Cape, there was limited leadership by facility managers.^32^ It was linked to a top-down policy that did not take into account the complexities of the health system, such as the dual structure of provincial and local government authority, with facility managers being accountable to local government authority mandate and district managers accountable to provincial structures.^57^ The challenging context included competing priorities where CHW programme was only one of the healthcare reforms being implemented at the local level.^58^

In another district in Gauteng Province and in parts of the Western Cape, the CHWs operated through health posts and NGOs rather than health facilities. Having the team separated from the health facilities resulted in better relationships with facility-based staff including facility managers. The CHW reported directly to subdistrict and district managers, thus minimising the complication of dual lines of reporting to local government and provincial authorities, as was the case in our study.^32^

The unintended consequences of inadequate preparation for the new policy at the local level included resistance of facility-based staff towards CHWs; as a result, WBPHCOTs did not feel integrated into the community health system.^59^ In addition, there was a lack of awareness about the programme in the community and the subsequent resistance of some households to the presence CHWs, which influenced their ability to perform their roles.

The preponderance of women in all staff categories in health facilities, but in particular CHWs, is a common gender imbalance in South Africa and globally.^18 60^ The CHWs undertook the care roles which were aligned to the gender roles which were normative in the community, with implications for their own health and finances.^16 60^

The lack of formal appointments of CHWs resulted from the cadre not being established within the human resources policy of the National Department of Health. CHWs were paid a stipend, which did not allow for differentiation based on experience or qualifications. The lack of formal recognition affected the conditions of employment, and there was no clear career path for CHWs. This finding was consistent with other research in the country and has been identified as a factor affecting motivation and retention^24 52 61–68^ and unlike the model plan from Brazil where CHWs were formalised as part of a job creation programme.^69^

Since the research was completed, the National Department of Health has revised the policy stating that enrolled nurses (a cadre with fewer skills and qualifications) should be team leaders of the WBPHCOTs rather than professional nurses as originally designed. The adaptation reflects one solution to the human resource challenges. Yet, our study suggests that this solution will only address one issue and that enrolled nurses lower-down in nursing hierarchy may have fewer skills to adequately support the CHWs.^24 70^ Furthermore, team leaders shouldered the responsibility of an expanded mandate including the complex task of working intersectorally, which other cadre of health workers in the health system struggle with.^71 72^

Similarly, the consequence of rushed implementation was the insufficient time to build trust and a shared vision with local community leaders in order to create partnerships supportive of the CHW programme.56 Building trust in the community could have offered some protection. CHWs in the district reported violent mugging, sexual assault and exposure to ‘nyaope’ users.^73 74^

Competency of CHWs and team leaders to perform as expected was compromised by training deficiencies and absence of ongoing supportive supervision.^7 13 16 17 75–78^ Enrolled nurses themselves felt unskilled because of the short duration of training and lack of PHC expertise. The 10-day training for CHWs is very short compared with that in other countries such as Brazil where CHWs receive an 8-week residential course, 4 weeks of fieldwork (practical) followed by on-going training session.^5^ Practical training is essential for CHWs to learn new knowledge but more importantly develops both skill and confidence.^79^ We found that only one team had received practical training, despite being in place for at least 6 months.

Learning occurred beyond the formal training through peer support. CHWs completed some tasks collectively, which allowed the CHWs with more capacity to share skills with those who had less competencies; this initiative contributed to programme implementation. The phenomenon of peer support learning has been noted in other contexts, where CHWs mobilised existing social capital within the community.^16 24 80^

### Limitation of the ISF

While the ISF recognises that change is not a linear process but more cyclic in nature, it demarcates different stages which suggest that there are a set of processes and activities that are rational and ordered. These assumptions do not always correspond to real-world experiences. In other words, the ISF does not take sufficiently into account complexity such as the messiness of local actor contexts and ecosystems of implementation with its multiple interactions, local norms and micropolitics as reflected in the results. Another limitation of the ISF is that it does not account for the “how” and the “why” factors affecting implementation. It is tied to a methodological limitation where data were collected from actors who are implementers (those running the programme) rather than managers who are making key implementation decisions.

While helpful, the ISF does not adequately take into consideration the multilevel sensitive approaches required for whole system change, such as required in community health system contexts.^33^ In addition, it does not sufficiently consider how micropractices of power exercised by front-line managers influence the experience and consequences of policy implementation.^51 81 82^ It might be useful in later studies to consider how complex adaptive health systems intersect with implementation science to achieve equity and social justice goals.^42^

## Conclusion

The implementation of complex programmes is a challenge. Re-engineering of PHC was a policy advance, but its lack of detail on practice with respect to resources, budget, supervision, training and sustainability through the continued commitment of CHW made a difficult furrow to plough. Policy implementation requires shared vision on policy intentions, detailed initial central planning and practical ways to provide ongoing support and a feedback loop to share learnings and adaptations that occur at the lower level to other settings. This study points to how progressive reform remains fraught without due attention to the minutiae of practice.
